# Supplementary material for: Bio-inspired cryptography based on proteinoid assemblies
Source: PLoS One. 2025 May 28;20(5):e0324761. doi: 10.1371/journal.pone.0324761 (PMC12118996; doi:10.1371/journal.pone.0324761)
Supplement: Attachment — Submitted filename: Response_to_Reviewers.pdf [file pone.0324761.s001.pdf]

CC: daniel.hunyadi@ulbsibiu.ro

PONE-D-24-49897

Bio-Inspired Cryptography Based on Proteinoid Assemblies

PLOS ONE

Dear Dr. Mougkogiannis,

Thank you for submitting your manuscript to PLOS ONE. After careful consideration, we feel that it has merit but does not fully meet PLOS ONE's publication criteria as it currently stands. Therefore, we invite you to submit a revised version of the manuscript that addresses the points raised during the review process.

Please submit your revised manuscript by Jan 12 2025 11:59PM. If you will need more time than this to complete your revisions, please reply to this message or contact the journal office at plosone@plos.org. When you're ready to submit your revision, log on to https://www.editorialmanager.com/pone/ and select the 'Submissions Needing Revision' folder to locate your manuscript file.

Please include the following items when submitting your revised manuscript:

- **A rebuttal letter that responds to each point raised by the academic editor and reviewer(s). You should upload this letter as a separate file labeled 'Response to Reviewers'.**
- **A marked-up copy of your manuscript that highlights changes made to the original version. You should upload this as a separate file labeled 'Revised Manuscript with Track Changes'.**
- **An unmarked version of your revised paper without tracked changes. You should upload this as a separate file labeled 'Manuscript'.**

If you would like to make changes to your financial disclosure, please include your updated statement in your cover letter. Guidelines for resubmitting your figure files are available below the reviewer comments at the end of this letter.

If applicable, we recommend that you deposit your laboratory protocols in protocols.io to enhance the reproducibility of your results. Protocols.io assigns your protocol its own identifier (DOI) so that it can be cited independently in the future. For instructions see: https://journals.plos.org/plosone/s/submission-guidelines#loc-laboratory-protocols. Additionally, PLOS ONE offers an option for publishing peer-reviewed Lab Protocol articles, which describe protocols hosted on protocols.io. Read more information on sharing protocols at https://plos.org/protocols?utm_medium=editorial-email&utm_source=authorletters&utm_

campaign=protocols.

We look forward to receiving your revised manuscript.

Kind regards,

Daniel Ioan Hunyadi, Ph.D.
Academic Editor
PLOS ONE

Journal Requirements:

When submitting your revision, we need you to address these additional requirements.

1. Please ensure that your manuscript meets PLOS ONE's style requirements, including those for file naming. The PLOS ONE style templates can be found at

https://journals.plos.org/plosone/s/file?id=wjVg/PLOSOne_formatting_sample_main_body.pdf and

https://journals.plos.org/plosone/s/file?id=ba62/PLOSOne_formatting_sample_title_authors_affiliations.pdf

2. Please note that PLOS ONE has specific guidelines on code sharing for submissions in which author-generated code underpins the findings in the manuscript. In these cases, we expect all author-generated code to be made available without restrictions upon publication of the work. Please review our guidelines at https://journals.plos.org/plosone/s/materials-and-software-sharing#loc-sharing-code and ensure that your code is shared in a way that follows best practice and facilitates reproducibility and reuse.

3. Thank you for stating the following financial disclosure: [y EPSRC Grant EP/W010887/1 “Computing with proteinoids”]. Please state what role the funders took in the study. If the funders had no role, please state: "The funders had no role in study design, data collection and analysis, decision to publish, or preparation of the manuscript." If this statement is not correct you must amend it as needed. Please include this amended Role of Funder statement in your cover letter; we will change the online submission form on your behalf.

4. Please expand the acronym “EPSRC” (as indicated in your financial disclosure) so that it states the name of your funders in full. This information should be included in your cover letter; we will change the online submission form on your behalf.

5. Please amend your list of authors on the manuscript to ensure that each author is linked to an affiliation. Authors’ affiliations should reflect the institution where the work was done (if authors moved subsequently, you can also list the new affiliation stating

“current affiliation:….” as necessary).

[Note: HTML markup is below. Please do not edit.]

Reviewers' comments:

Reviewer's Responses to Questions

Comments to the Author

1. Is the manuscript technically sound, and do the data support the conclusions?

The manuscript must describe a technically sound piece of scientific research with data that supports the conclusions. Experiments must have been conducted rigorously, with appropriate controls, replication, and sample sizes. The conclusions must be drawn appropriately based on the data presented.

Reviewer #1: Partly

Reviewer #2: Yes

---

2. Has the statistical analysis been performed appropriately and rigorously?

Reviewer #1: No

Reviewer #2: I Don't Know

---

3. Have the authors made all data underlying the findings in their manuscript fully available?

The PLOS Data policy requires authors to make all data underlying the findings described in their manuscript fully available without restriction, with rare exception (please refer to the Data Availability Statement in the manuscript PDF file). The data should be provided as part of the manuscript or its supporting information, or deposited to a public repository. For example, in addition to summary statistics, the data points behind means, medians and variance measures should be available. If there are restrictions on publicly sharing data—e.g. participant privacy or use of data from a third party—those must be specified.

The data is available from the Zenodo database here: https://zenodo.org/records/13924338

Reviewer #1: No

Reviewer #2: Yes

---

4. Is the manuscript presented in an intelligible fashion and written in standard English?

PLOS ONE does not copyedit accepted manuscripts, so the language in submitted articles must be clear, correct, and unambiguous. Any typographical or grammatical errors should be corrected at revision, so please note any specific errors here.

Reviewer #1: Yes

Reviewer #2: Yes

---

5. Review Comments to the Author

Please use the space provided to explain your answers to the questions above. You may also include additional comments for the author, including concerns about dual publication, research ethics, or publication ethics. (Please upload your review as an attachment if it exceeds 20,000 characters)

Reviewer #1: Title- Bio-Inspired Cryptography Based on Proteinoid Assemblies
1. Justify the statement " Bioinspired cryptography has emerged as a significant area of research,".

This emerging field uses biological principles that have evolved over millions of years to solve complex security problems~\cite{skormin2001basis,mthunzi2019bio}. Biological systems have strong data protection methods~\cite{xiao2006new}. They can repair DNA defects, detect immune systems, and learn from evolutionary networks~\cite{harmer2002artificial}. This approach lays a strong foundation for new algorithms. They can be more secure, efficient, and scalable than traditional methods. This field is important. It can solve challenges using biologically proven solutions~\cite{shandilya2023nature}. This is especially true for key generation, authentication, and secure communication protocols~\cite{sarkar2020novel,xi2011fingerprint,panchal2018novel}.

2. The table 1 compare the bio inspired cryptography and key based cryptography, what is the need of both of the approach.

Table~\ref{tab:biological-encryption} compares biologically inspired cryptography techniques. Each has its own benefits but supports traditional cryptography. Traditional

cryptography uses math and established protocols. Biologically inspired methods use new techniques from biology to boost security. DNA-based cryptography uses the vast power of DNA's parallel processing. It also uses traditional key functions. Cellular automata-based methods create complex, unpredictable patterns. These can enhance existing cryptographic primitives. This complementary relationship lets systems use both methods. They get the core security of traditional approaches and the new abilities of bionic ones. This results in deep cryptographic hybrid solutions.

3. materials and methods section is too small, i have a suggestion to add more data from the result section.

Dear Reviewer,

Thank you for your comments. We have added the MATLAB code methodology for generating PSD plots in the Methods section.

Thanks,

Reviewer #2: This paper seems to have some innovative scientific crypto contribution benefiting from deterministic interactions of proteinoid assemblies. The work generates secure encryption keys for algorithms using unpredictability of proteinoid behaviour that is considered adaptable encryption framework resisting known cryptanalytic attacks. The exploration illustrates the implementation of bio-inspired system based on simulated proteinoid interactions resulting promissing encryption strength. The research show random key generation presenting security of standard statistical tests immune to classical and quantum computers attacks. This study innovates security via biological principles of potential processes in cryptography needing overall secrecy reconsideration in order to be in ready and complete. Therefore, the following secrecy references are recommended to be involved:

== “AI Based Mobile Edge Computing for IoT: Applications, Challenges, and Future Scope”, Arabian Journal for Science and Engineering (2022). http://doi.org/10.1007/s13369-021-06348-2

== "Personal Privacy Evaluation of Smart Devices Applications Serving Hajj and Umrah Rituals", Journal of Engineering Research (2021). http://doi.org/10.36909/jer.13199

== "Cybercrime on Transportation Airline", Journal of Forensic Research, ISSN: 2157-7145, 10(4):449 (2019)

== "Sensing e-Banking Cybercrimes Vulnerabilities via Smart Information Sciences Strategies", RAS Engineering and Technology 1(1):1-9 (2020)

== "Engineering Graphical Captcha and AES Crypto Hash Functions for Secure Online Authentication", Journal of Engineering Research (2021). http://doi.org/10.36909/jer.13761

The work is more interesting if considering the following image hiding strategies proposed within below encryption researches:
== "Varying PRNG to improve image cryptography implementation", Journal of Engineering Research 9(3A):153-183 (2021).
== "Remodeling Randomness Prioritization to Boost-up Security of RGB Image Encryption", Multimedia Tools and Applications (MTAP) 80(18):28521–28581 (2021)
== “Dynamic smart random preference for higher medical image confidentiality”, Journal of Engineering Research (2022) http://doi.org/10.36909/jer.17853
== "Improving data hiding within colour images using hue component of HSV colour space", CAAI Transactions on Intelligence Technology, 7(1): 56–68 (2022)
== "Efficient Image Reversible Data Hiding Technique Based on Interpolation Optimization", Arabian Journal for Science and Engineering (AJSE) 46(9):8441–8456 (2021)
== "Efficient Reversible Data Hiding Multimedia Technique Based on Smart Image Interpolation", Multimedia Tools and Applications (MTAP) 79(39):30087-30109 (2020)

Dear Reviewer,

Thank you for your insightful comments. Following your recommendation, I have added the suggested references to our manuscript.

---

6. PLOS authors have the option to publish the peer review history of their article (what does this mean?). If published, this will include your full peer review and any attached files.

If you choose “no”, your identity will remain anonymous but your review may still be made public.

Do you want your identity to be public for this peer review? For information about this choice, including consent withdrawal, please see our Privacy Policy.

Reviewer #1: Yes: Sultan H. Almotiri

Reviewer #2: No
