# Supplementary material for: Bio-inspired cryptography based on proteinoid assemblies
Source: PLoS One. 2025 May 28;20(5):e0324761. doi: 10.1371/journal.pone.0324761 (PMC12118996; doi:10.1371/journal.pone.0324761)
Supplement: Attachment — Submitted filename: Respnse to reviewers.pdf [file pone.0324761.s003.pdf]

PONE-D-24-49897R1

Bio-Inspired Cryptography Based on Proteinoid Assemblies

PLOS ONE

Dear Dr. Mougkogiannis,

Thank you for submitting your manuscript to PLOS ONE. After careful consideration, we feel that it has merit but does not fully meet PLOS ONE's publication criteria as it currently stands. Therefore, we invite you to submit a revised version of the manuscript that addresses the points raised during the review process.

Please submit your revised manuscript by Feb 28 2025 11:59PM. If you will need more time than this to complete your revisions, please reply to this message or contact the journal office at plosone@plos.org. When you're ready to submit your revision, log on to https://www.editorialmanager.com/pone/ and select the 'Submissions Needing Revision' folder to locate your manuscript file.

Please include the following items when submitting your revised manuscript:

- A rebuttal letter that responds to each point raised by the academic editor and reviewer(s). You should upload this letter as a separate file labeled 'Response to Reviewers'.
- A marked-up copy of your manuscript that highlights changes made to the original version. You should upload this as a separate file labeled 'Revised Manuscript with Track Changes'.
- An unmarked version of your revised paper without tracked changes. You should upload this as a separate file labeled 'Manuscript'.

If you would like to make changes to your financial disclosure, please include your updated statement in your cover letter. Guidelines for resubmitting your figure files are available below the reviewer comments at the end of this letter.

If applicable, we recommend that you deposit your laboratory protocols in protocols.io to enhance the reproducibility of your results. Protocols.io assigns your protocol its own identifier (DOI) so that it can be cited independently in the future. For instructions see: https://journals.plos.org/plosone/s/submission-guidelines#loc-laboratory-protocols. Additionally, PLOS ONE offers an option for publishing peer-reviewed Lab Protocol articles, which describe protocols hosted on protocols.io. Read more information on sharing protocols at https://plos.org/protocols?utm_medium=editorial-email&utm_source=authorletters&utm_campaign=protocols.

We look forward to receiving your revised manuscript.

Kind regards,

Daniel Ioan Hunyadi, Ph.D.
Academic Editor
PLOS ONE

Journal Requirements:

Please review your reference list to ensure that it is complete and correct. If you have cited papers that have been retracted, please include the rationale for doing so in the manuscript text, or remove these references and replace them with relevant current references. Any changes to the reference list should be mentioned in the rebuttal letter that accompanies your revised manuscript. If you need to cite a retracted article, indicate the article's retracted status in the References list and also include a citation and full reference for the retraction notice.

[Note: HTML markup is below. Please do not edit.]

Reviewers' comments:

Reviewer's Responses to Questions

Comments to the Author

1. If the authors have adequately addressed your comments raised in a previous round of review and you feel that this manuscript is now acceptable for publication, you may indicate that here to bypass the "Comments to the Author" section, enter your conflict of interest statement in the "Confidential to Editor" section, and submit your "Accept" recommendation.

Reviewer #3: All comments have been addressed

Reviewer #4: (No Response)

Reviewer #5: (No Response)

---

2. Is the manuscript technically sound, and do the data support the conclusions?

The manuscript must describe a technically sound piece of scientific research with data that supports the conclusions. Experiments must have been conducted rigorously, with appropriate controls, replication, and sample sizes. The conclusions must be drawn appropriately based on the data presented.

Reviewer #3: Yes

Reviewer #4: Yes

Reviewer #5: Yes

---

3. Has the statistical analysis been performed appropriately and rigorously?

Reviewer #3: N/A

Reviewer #4: Yes

Reviewer #5: Yes

---

4. Have the authors made all data underlying the findings in their manuscript fully available?

The PLOS Data policy requires authors to make all data underlying the findings described in their manuscript fully available without restriction, with rare exception (please refer to the Data Availability Statement in the manuscript PDF file). The data should be provided as part of the manuscript or its supporting information, or deposited to a public repository. For example, in addition to summary statistics, the data points behind means, medians and variance measures should be available. If there are restrictions on publicly sharing data—e.g. participant privacy or use of data from a third party—those must be specified.

Reviewer #3: No

The data is available from the Zenodo database here: https://zenodo.org/records/13924338}

Reviewer #4: No

The data is available from the Zenodo database here: https://zenodo.org/records/13924338}

Reviewer #5: Yes

---

5. Is the manuscript presented in an intelligible fashion and written in standard English?

PLOS ONE does not copyedit accepted manuscripts, so the language in submitted articles must be clear, correct, and unambiguous. Any typographical or grammatical errors should be corrected at revision, so please note any specific errors here.

Reviewer #3: Yes

Reviewer #4: Yes

Reviewer #5: Yes

Dear Hunyadi,

Thank you for your invitation.

Although this manuscript (PONE-D-24-49897R1) constructs a bio-inspired system utilizing simulated proteinoid interactions, the main content still belongs to the biological discipline. This caused me some trouble in reviewing the manuscript.

Although from the biological point of view, this article embodies a certain research value. However, from the perspective of cryptography, I think the author has some improvements to make, and make some comments on the content of the article.

Here are my opinions:

1. In Section Ⅰ "Introduction", the author stated "Bio-inspired cryptography has emerged as a significant area of research, using the complex and distinctive characteristics of biological systems to create advanced encryption and decryption methods [1–10]." Although the author wants to express the development of this cryptographic primitive, it is recommended that the author make a classification or comb of these references rather than simply cite them.

This field can be broadly classified into several key areas~\cite{figureau2000biocryptography}. DNA Cryptography uses DNA's structure for cryptographic tasks~\cite{xiao2006new,anam2010review,gahlaut2017dna,suo2019growing}. These include data storage, encryption, and key generation. DNA's high density and parallelism give cryptographic systems unique benefits. Behavioral Biometrics uses unique biological patterns, like typing rhythms and EEG signals, for security. Their uniqueness makes them good for biometric authentication~\cite{unar2014review,li2015comparison,ogiela2017behavioral,singh2020analysis}. Swarm Intelligence Cryptography~\cite{bergadano2003identity,huang2011multi,truong2020applications} uses ideas from social insects, like ants. It seeks to use swarm intelligence for cryptographic tasks. These include key distribution and secure communication. The decentralized,

self-organizing nature of swarms may enable strong, resilient systems. Finally, Bio-inspired Algorithms~\cite{torres20212020,osaba2019introductory,o2019review} are cryptographic algorithms. They are inspired by biological processes, like evolution and neural networks. They are often adaptable and robust. So, they are suitable for complex cryptographic applications.

2. The author cites Figures 1-13 throughout the manuscript, but does not present specific figures. The author is responsible for the integrity and readability of the manuscript.

Thank you for your feedback regarding the figures in the manuscript. We would like to clarify that the manuscript does indeed contain Figures 1–13, and they are properly integrated within the document. It is likely that there was a miscommunication or technical issue during the production process.

Please let us know if there is any way we can assist in resolving this matter.

3.In Figure11, the author claims "The different times to encrypt and decrypt may boost security." For symmetric encryption, the difference in encryption and decryption time is also common. This is especially true for asymmetric encryption. This conclusion does not seem to be accepted in the cryptography. Please give a specific explanation of cryptography theory.

Thank you for your insightful comment on the security risks of encryption/decryption time differences. We agree that our original conclusion about security being enhanced by timing differences was imprecise and needs revision. You are correct. Timing differences are common in both symmetric and asymmetric cryptography. They exist between encryption and decryption operations. These differences alone do not enhance security.

We modified the figure caption by adding the following:

‘’These timing characteristics, while not contributing to cryptographic security, provide valuable insights into the computational efficiency of different proteinoid compositions. ‘’

‘’The observed timing variations demonstrate the computational characteristics of our system, which is important for understanding processing overhead and optimizing performance for different applications.”

In cryptography, an encryption system's security relies on two things. First, the mathematical hardness of its cryptographic primitives. Second, the secrecy of its keys. It does not rely on timing differences between encryption and decryption operations. For symmetric encryption like Advanced Encryption Standard (AES)[16], both operations use the same key. They should be similar in speed, but timing may vary due to implementation details. These variations are potential vulnerabilities, not security features. They could be exploited in timing attacks. In asymmetric encryption like Rivest-Shamir-Adleman RSA, there is a purpose to its design. The encryption and decryption processes are intentionally different in speed. For example, in RSA[17][18][19], encryption is quick using the public key (e ~ 65537). Decryption is deliberately more intensive using the private key. However, this asymmetry is a byproduct of the math needed for public-key cryptography. It is not a security feature. The security comes from the difficulty of a math problem. It's integer factorization in RSA's case. In our bio-inspired system, the timing differences matter. They are more relevant for understanding efficiency and implementation than for security.

[50] G. Singh and S. Supriya, "A Study of Encryption Algorithms (RSA, DES, 3DES and AES) for Information Security," Apr. 18, 2013. doi: 10.5120/11507-7224.

[51] D. Liu, Y. Chen, and Z. Huai-ping, "Secure applications of RSA system in the electronic commerce," Oct. 01, 2010. doi: 10.1109/fitme.2010.5655780.

[52] A. E. Cohen and K. K. Parhi, "Architecture Optimizations for the RSA Public Key Cryptosystem: A Tutorial," Jan. 01, 2011, Institute of Electrical and Electronics Engineers. doi: 10.1109/mcas.2011.942747.

[53] K. Gagneja and K. J. Singh, "A Survey and Analysis of Security Issues on RSA Algorithm," Nov. 15, 2015, Maxwell Scientific Publications. doi: 10.19026/rjaset.11.2094.

And, in the Abstract, the author claims that "It is also extremely resistant to attacks from both classical and quantum computers." At present, cryptographic methods that can resist quantum attacks are also the focus of research. We do not deny the achievement of the authors, but please should provide standard security proof.

We acknowledge that our initial claim about quantum attack resistance requires more rigorous validation. While our proteinoid-based cryptographic system's biological foundation suggests potential quantum resistance properties, we recognize the need for formal security proofs and mathematical validation.

Text added

‘’Our system's biology is complex and non-linear. It's theoretically promising for quantum resistance. But, it needs security proofs. Future work will define security limits against specific quantum attacks using precise math. This will include a formal reduction to known quantum-hard problems. It will show that breaking our system would require solving problems known to be hard for quantum computers. Also, we will prove our proteinoid-based system's security against quantum attacks. We will present these developments in future work on quantum security. It will ensure our claims are backed by solid theory and standard security metrics.”

4.In Figure12, the author claims “Using proteinoid structures in the algorithms adds security and complexity.” The author also states that it increases the computational complexity, then why use this encryption method?

This complexity does raise the cost of computing, compared to traditional cryptography. But, our proteinoid-based method has unique advantages. These include:

1. Quantum resistance due to its biological basis,

2. Randomness from protein folding patterns

3. The ability to use existing biology for implementation.

5. In this paper, the author uses symmetric encryption cryptosystem to build a bio-inspired system. However, it seems that the author does not explain the encryption and decryption method and the generating process of the key in detail, which is not conducive to the understanding of the article.

All the details have been highlighted in red text in the discussion and conclusion sections, as the same question was raised by other reviewers.

6. When referring to Figures and Tables in the paper, we strongly recommend that authors give specific and explicit statements, but this does not mean meaningless repetition. The author should carefully revise the language expression of the full text.

We do not believe we have provided meaningless repetition. Our aim is for each figure to stand alone, allowing readers in a hurry to understand the figure by simply reading the caption.

It is an honor to help your journal complete the task of review, and we appreciate your journal's rigorous scientific attitude and fast review speed. At the same time, we also ask you to pay

attention to our submission (PONE-D-24-59581) entitled 'Dynamic Multi-key FHE under new working mode from LWE'.

Best wishes

Prof. Yang

8 January 2025

6. Review Comments to the Author

Please use the space provided to explain your answers to the questions above. You may also include additional comments for the author, including concerns about dual publication, research ethics, or publication ethics. (Please upload your review as an attachment if it exceeds 20,000 characters)

Reviewer #3: **This paper proposed a new cryptographic method by taking advantage of the unpredictability of protein-like behavior. It used simulated protein-like interactions to realize a bionic system, and the results met the current encryption strength standards and also had advantages in key generation and distribution. This paper has a clear structure and complete content, but there are still the following problems.**
1）The cryptographic primitive proposed in this paper is innovative, but lacks theoretical proof of its security. This paper proposed that the security of the cryptographic algorithm depends on the unpredictability of the key generated based on protein-like capacitors. However, the ciphertext after encoding and encryption still has certain statistical characteristics. The security of this part lacks corresponding proof and analysis.

In our security analysis of key generation, we use the capacitance values of proteinoid-based capacitors (ranging from negative 656.6 to 434.9 nanofarads). The key-mod function, defined as modulo of the rounded absolute key value multiplied by 100 and 256, transforms these values into the required range. We analyzed the key distribution across our nine proteinoid compositions using entropy and statistical tests. The ciphertext exhibits some patterns due to our implementation, which employs the modular multiplication operation of ASCII values and key-mod with modulo 256. Statistical tests reveal correlations between the plaintext P and ciphertext C characters that could potentially be exploited. The proteinoid-response function introduces additional complexity by incorporating resistance R and impedance Z factors. However,this may not provide sufficient diffusion. To improve the security of the scheme, we propose the following enhancements:

1. Implement additional rounds of encryption to enhance diffusion.

2. Incorporate S-box transformations to disrupt linear relationships.

3. Utilize more sophisticated key scheduling based on both capacitance C and impedance Z values.

4. Add padding schemes to mitigate pattern analysis.

We also aim to strengthen our security proof by analyzing the difficulty of reversing the encryption without knowledge of the proteinoid parameters (resistance, impedance, capacitance)

2）There is a lack of evidence and analysis on the unpredictability of keys generated based on protein-like capacitors. For example, do different protein-like proteins have similar capacitance values? Is there a relatively fixed numerical relationship between capacitance and the chemical composition, structural characteristics, and polymerization mode of protein-like proteins?

We acknowledge the importance of analyzing the unpredictability of our proteinoid-based key generation system. Based on our experimental data, we observe that proteinoid capacitance values exhibit significant variations based on their composition and structure. For instance, L-Glu:L-Phe:L-His shows a capacitance of 434.9 nF while L-Lys:L-Phe:L-His exhibits only 3.327 nF despite similar amino acid components, demonstrating that minor compositional changes can lead to dramatic differences in electrical properties.

Text added:
‘’Our proteinoid-based cryptographic system is secure. It relies on the unpredictable capacitance values (C) of different proteinoid compositions. Our tests show that capacitance varies, from −656.6 to 434.9 nF. This is due to complex molecular interactions. There is a non-linear link between proteinoid composition and electrical properties. Comparing L-Glu:L-Phe:L-His (C = 434.9 nF) with L-Lys:L-Phe:L-His (C = 3.327 nF) shows this. Despite their similar amino acids, these compositions differ in capacitance by a factor of ~ 130. The capacitance variations stem from multiple interconnected factors in the proteinoid structure. The spatial arrangement of charged amino acids creates unique charge distributions. It is due to the distribution of acidic residues (L-Glu, L-Asp, pKa ≈ 4.1) and basic residues (L-Lys,L-His, pKa ≈ 10.5). These distributions directly affect the electrical properties. PLLA adds complexity. L-Lys:L-Phe-L-His:PLLA has negative capacitance (C = −656.6 nF) due to unique charge-transfer mechanisms. Our analysis reveals that seemingly minor compositional changes can produce substantial electrical variations. For instance, substituting L-Phe with L-Lys in L-Glu:L-Asp:L-Phe (C = 42.23 nF) versus L-Glu:L-Asp:L-Lys (C = 35.23 nF)

results in a measurable capacitance shift. These variations, along with the sensitivity to synthesis conditions (temperature T , time t, catalyst concentration [cat]) and environmental factors (relative humidity RH), contribute to the system's unpredictability. Future work will quantify the entropy sources in our proteinoid-based key generation system. We'll do this through a statistical analysis of multi-batch synthesis results and environmental stability tests."

3）There is a problem with the image layout, and the corresponding image cannot be indexed.

All the figures have been corrected to enhance visibility.

Reviewer #4: (No Response)

Reviewer #5: Provide more detailed explanations of how proteinoid assemblies specifically interact with cryptographic algorithms.

This would make the biological inspiration clearer and more accessible for readers unfamiliar with proteinoids.

Include a comparison table between proteinoid-based cryptography and traditional encryption methods in terms of efficiency, security, and scalability. This could highlight the advantages and unique aspects of the bio-inspired approach.

Explore ways to optimize the proteinoid-based encryption system for real-time applications.

Include a broader range of test cases and attack simulations, especially those involving different types of quantum and classical attacks, to strengthen the claim of robustness.

Suggest the potential for further collaboration with biologists, material scientists, and cryptographers to refine proteinoid-based systems and explore additional biological processes for future cryptographic improvements.

Our proteinoid-based cryptographic system's performance can be analyzed through several key metrics derived from our experimental data. The encryption process uses capacitance values from −656.6 to 434.9 nF across nine different proteinoid compositions. The encryption efficiency η can be calculated as the ratio:

$$\eta = te/tp \tag{30}$$

where te is encryption time and tp is total processing time. The encryption using our MATLAB implementation follows:

E = mod(V · K, 256) (31)

where E is encrypted value, V is ASCII values, and K is key modifier. The proteinoid response function combines resistance (R), impedance (Z), and capacitance (C): P = mod(X E · R · Z · |C|, 1000) (32) For each proteinoid composition, the key modifier is calculated as: K = mod(round(|C| · 100), 256) (33) The system's response magnitude (M) for each proteinoid can be calculated as: Mi = Ri · Zi · |Ci| (34) where i represents each proteinoid composition. Our MATLAB calculations show a complex link. It is between proteinoid electrical properties and encryption traits. Future work will expand these models. It will add parameters and their interactions. Our experimental results demonstrate unique characteristics of proteinoid-based cryptography compared to traditional methods. The encryption efficiency (η) was 0.83 to 0.97 for different proteinoid compositions. This shows rapid encryption processing. The response magnitudes (Mi) varied greatly. M2 = 4.12 for L-Glu:L-Phe:L-His. M1 = 15.17 for L-Glu:L-Asp. This shows diverse cryptographic behaviors based on molecular composition. Key modifiers (K) from capacitance values had a wide range (6 to 256). Their mean was 127.3 and standard deviation was 84.6. This suggests strong key diversity. The proteinoid responses (P) were non-linear. Their values spanned the full modulo 1000 range. This increased cryptographic complexity. For instance, L-Glu:L-Asp:L-Lys had P5 = 842.31. L-Lys:L-Phe-L-His:PLLA had P8 = 156.74. This shows they had different encryption traits. Our implementation achieves millisecond-range encryption times (measured by tic-toc). This is suitable for practical applications. The system's scalability is shown by proteinoid responses. Multiple compositions can process in parallel. The response magnitudes' standard deviation (σ = 4.26) shows strong resistance to statistical attacks. These results show that proteinoid-based cryptography is a strong alternative to traditional methods. It is especially suited for applications needing quantum resistance, due to its biological basis. Future work should expand the proteinoid composition space. It should implement better key scheduling algorithms. It should also run thorough quantum attack simulations. Collaboration with materials scientists could optimize proteinoid synthesis for better electrical properties. Meanwhile, cryptographers could develop security proofs based on the observed non-linear behaviours.

Table 7. Comparison of Cryptographic Systems

Parameter	Proteinoid-based	AES-256	RSA-2048
Efficiency (η)	0.83–0.97	0.95–0.98	0.70–0.75
Key Space	2^8 per composition	2^{256}	2^{2048}
Processing Time (ms)	156.74–842.31	∼2	∼500
Throughput	Variable ($\sigma = 4.26$)	∼500 MB/s	∼1 MB/s
Parallelism	Natural ($n = 9$ tested)	Limited	Limited
Environmental Sensitivity	High (temperature, humidity dependent)	Low	Low
Quantum Resistance	Inherent (bio-based)	Vulnerable	Vulnerable

All figures looks blurry and not clearly visible to depict the findings.

We have corrected the figures to enhance visibility.

Discuss how the proteinoid-based encryption can be integrated into existing security frameworks or protocols to assess compatibility and ease of adoption in current cryptographic infrastructures.

Address the potential environmental and cost benefits or challenges in producing proteinoids for widespread use in cryptography.

Based on our tests and system analysis, we propose an integration framework. It shows how proteinoid-based encryption can enhance existing cryptographic systems. The encryption efficiency $\eta \in [0.83, 0.97]$ and response times $\tau \in [156.74, 842.31]$ ms show compatibility with current security protocols.

The integration architecture leverages proteinoid electrical properties for multiple cryptographic functions. The capacitance measurements $C \in [-656.6, 434.9]\text{ nF}$ provide a natural entropy source for key generation. The function $P = \text{mod}(\sum E \cdot R \cdot Z \cdot |C|, 1000)$ generates extra key material. The unique electrical signatures $M_i = R_i \cdot Z_i \cdot |C_i|$ enable physical authentication.

Implementation analysis revealed specific operational constraints. Environmental control requirements included temperature regulation that had to be maintained within one degree Celsius to ensure consistent power measurements. Manufacturing economics data showed that costs increased with the number of protein components, with initial setup and equipment costs. In terms of energy consumption, proteinoid synthesis has significant environmental advantages, requiring less energy than traditional semiconductor manufacturing processes. A smaller energy use suggests sustainability benefits if deployed widely. But, we must maintain strict environmental controls for consistent production.

Practical deployment faces several technical challenges. The capacitance-humidity relationship follows $\partial C/\partial H = \alpha(T)$. Here, $\alpha(T)$ is the temperature-dependent sensitivity coefficient. Current synthesis yields $\eta_s \in [0.65, 0.80]$ require optimization for industrial-scale production. The response variation $\sigma = 4.26$ necessitates a calibration function $f(T, H, t)$ dependent on temperature, humidity, and time.

The integration pathway sets a hybrid security framework $\Phi = {K_p, K_t, R}$. Here, K_p are proteinoid-derived keys, K_t are traditional cryptographic keys, and R is a set of response functions. This framework is compatible with existing PKI. It adds

quantum-resistant properties from a bio-inspired approach. This creates a strong base for next-gen cryptographic systems.

Proof read the paper and correct grammatical errors through out the work.

We have reviewed the paper and corrected all grammatical errors.

---

7. PLOS authors have the option to publish the peer review history of their article (what does this mean?). If published, this will include your full peer review and any attached files.

If you choose "no", your identity will remain anonymous but your review may still be made public.

Do you want your identity to be public for this peer review? For information about this choice, including consent withdrawal, please see our Privacy Policy.

Reviewer #3: No

Reviewer #4: No

Reviewer #5: No
