# Supplementary material for: Bio-inspired cryptography based on proteinoid assemblies
Source: PLoS One. 2025 May 28;20(5):e0324761. doi: 10.1371/journal.pone.0324761 (PMC12118996; doi:10.1371/journal.pone.0324761)
Supplement: Attachment — Submitted filename: PONE-D-24-49897R2.pdf [file pone.0324761.s004.pdf]

PONE-D-24-49897R2

Bio-Inspired Cryptography Based on Proteinoid Assemblies

PLOS ONE

Dear Dr. Mougkogiannis,

Thank you for submitting your manuscript to PLOS ONE. After careful consideration, we feel that it has merit but does not fully meet PLOS ONE's publication criteria as it currently stands. Therefore, we invite you to submit a revised version of the manuscript that addresses the points raised during the review process.

Please submit your revised manuscript by Mar 26 2025 11:59PM. If you will need more time than this to complete your revisions, please reply to this message or contact the journal office at plosone@plos.org. When you're ready to submit your revision, log on to https://www.editorialmanager.com/pone/ and select the 'Submissions Needing Revision' folder to locate your manuscript file.

Please include the following items when submitting your revised manuscript:

- A rebuttal letter that responds to each point raised by the academic editor and reviewer(s). You should upload this letter as a separate file labeled 'Response to Reviewers'.
- A marked-up copy of your manuscript that highlights changes made to the original version. You should upload this as a separate file labeled 'Revised Manuscript with Track Changes'.
- An unmarked version of your revised paper without tracked changes. You should upload this as a separate file labeled 'Manuscript'.

If you would like to make changes to your financial disclosure, please include your updated statement in your cover letter. Guidelines for resubmitting your figure files are available below the reviewer comments at the end of this letter.

If applicable, we recommend that you deposit your laboratory protocols in protocols.io to enhance the reproducibility of your results. Protocols.io assigns your protocol its own identifier (DOI) so that it can be cited independently in the future. For instructions see: https://journals.plos.org/plosone/s/submission-guidelines#loc-laboratory-protocols. Additionally, PLOS ONE offers an option for publishing peer-reviewed Lab Protocol articles, which describe protocols hosted on protocols.io. Read more information on sharing protocols at https://plos.org/protocols?utm_medium=editorial-email&utm_source=authorletters&utm_campaign=protocols.

We look forward to receiving your revised manuscript.

Kind regards,

Daniel Ioan Hunyadi, Ph.D.
Academic Editor
PLOS ONE

[Note: HTML markup is below. Please do not edit.]

Reviewers' comments:

Reviewer's Responses to Questions

**Comments to the Author**

1. If the authors have adequately addressed your comments raised in a previous round of review and you feel that this manuscript is now acceptable for publication, you may indicate that here to bypass the “Comments to the Author” section, enter your conflict of interest statement in the “Confidential to Editor” section, and submit your "Accept" recommendation.

Reviewer #6: All comments have been addressed

Reviewer #7: All comments have been addressed

---

2. Is the manuscript technically sound, and do the data support the conclusions?

The manuscript must describe a technically sound piece of scientific research with data that supports the conclusions. Experiments must have been conducted rigorously, with appropriate controls, replication, and sample sizes. The conclusions must be drawn appropriately based on the data presented.

Reviewer #6: No

Reviewer #7: No

---

3. Has the statistical analysis been performed appropriately and rigorously?

Reviewer #6: No

Reviewer #7: No

---

4. Have the authors made all data underlying the findings in their manuscript fully available?

The PLOS Data policy requires authors to make all data underlying the findings described in their manuscript fully available without restriction, with rare exception (please refer to the Data Availability Statement in the manuscript PDF file). The data should be provided as part of the manuscript or its supporting information, or deposited to a public repository. For example, in addition to summary statistics, the data points behind means, medians and variance measures should be available. If there are restrictions on publicly sharing data—e.g. participant privacy or use of data from a third party—those must be specified.

Reviewer #6: No

Reviewer #7: No

---

5. Is the manuscript presented in an intelligible fashion and written in standard English?

PLOS ONE does not copyedit accepted manuscripts, so the language in submitted articles must be clear, correct, and unambiguous. Any typographical or grammatical errors should be corrected at revision, so please note any specific errors here.

Reviewer #6: No

Reviewer #7: No

---

6. Review Comments to the Author

Please use the space provided to explain your answers to the questions above. You may also include additional comments for the author, including concerns about dual publication, research ethics, or publication ethics. (Please upload your review as an attachment if it exceeds 20,000 characters)

**Reviewer #6**: **In principle, a process that is not replicable in the same way, with the same values is random and one that generates uncorrelated output values, but which can be replicated by knowing some parameters of the generation algorithm, will produce a pseudorandom set. From the description made, the primary model from which the authors start could be one used for random systems. The applicability would be limited by the transmission model of these data sets, called seeds in cryptography, and used within pseudorandom models, as initial values of the system.**

**To get out of this scope of applicability, which does not allow the correlation of the**

emitter and the receiver in order to fulfill the fundamental axiom of a cryptographic system, the authors should illustrate the completeness of the proposed model. In this sense, it would be useful:

1. A dedicated section to clearly illustrate how the correlation of keys from the sender of the encryption data system to the receiver of the encrypted data is achieved, in order to fulfill d(e(m))=m?
2. A clear and mathematically substantiated explanation for a correlative question with the applicability of the proposed solution: why do the electrical properties of proteinoids provide mathematical security comparable to traditional methods (AES, RSA, etc.)?
3. How is it ensured that an attacker cannot replicate a set of proteinoids and generate the same keys?
4. The key generation algorithm is based on measurable physical values, but experimental variability could affect the reproducibility of the keys. How is the synchronization of the two parties involved in communication achieved? And in this sense, the correlation of these explanations with point 3.

Authors:

Thanks for your thoughtful review! Your excellent questions touch on key parts of our cryptographic approach. We agree that we need to clarify the link between the sender and receiver. This includes understanding mathematical security, protection from replication attacks, and key reproducibility. We added a new section called "Cryptographic Security Analysis." It explains each concern in detail.

‘’3.4 Cryptographic Security Analysis

This section looks at important parts of our cryptographic system. We will look at important correlation mechanisms. We’ll also explore the math behind security. Additionally, we will discuss replication resistance and synchronization protocols.

3.4.1 Key Correlation Between Sender and Receiver

Our proteinoid-based cryptographic system uses symmetric encryption. Here, both parties share the same proteinoid assemblies that have matching electrical properties. This protocol establishes the link between encryption and decryption, where d(e(m)) =m

Proteinoid Synthesis Correlation: A master set of proteinoid assemblies is made under strict conditions. The temperature is set to 180 ± 1 ◦C. It takes 24 ± 0.5

hours. The relative humidity is 40 ± 5%. We created many identical sets concurrently to ensure correlation.

- Key Distribution Protocol: Before communication, the sender and receiver share the same proteinoid samples via a secure channel. This exchange is our system's main way to distribute keys. It's like quantum key distribution but uses biological materials.
- Electrical Property Measurement: Both parties measure the capacitance (C), resistance (R), and impedance (Z) of their proteinoid samples. They use calibrated equipment, specifically the BK Precision LCR meter Model 891, which operates at a frequency of 300 kHz.
- Parameterized Key Generation: We derive the encryption/decryption key $k_i$ deterministically from these measurements:

$$k_i = (\lfloor |C_i| \cdot 100 \rfloor \bmod 256)$$

- Synchronized Decryption:The receiver uses the same function to derive the identical key:

$$d(e(m_j, k_i), k_i) = d(m_j \cdot k_i) \bmod 256, k_i = m_j$$

This symmetric system means that if the proteinoid samples match, both sides can create the same encryption and decryption keys on their own. This meets the key requirement: $d(e(m)) = m$

### 3.4.2 Mathematical Security Foundations

Our proteinoid-based approach is secure. It compares well to traditional cryptographic methods due to several key mathematical properties. Our encryption function

$$e_j = m_j \cdot k_i \bmod 256$$

adds non-linearity with modular multiplication. This is a key element in many secure

cryptosystems, like AES. A single proteinoid sample offers a key space of 28. However, we can greatly boost our system's security in different ways. Multiple proteinoid samples can be combined to create composite keys:

$K_{composite} = n M_{i=1} k_i$.

Time-varying keys can be derived by measuring changes in electrical properties over time:

$k_i(t) = (\lfloor |C_i(t)| \cdot 100 \rfloor \bmod 256)$ .

Additional electrical parameters can increase the key space. We express this as:

$k_{expanded} = H R_i \parallel Z_i \parallel C_i$.

Here, H is a cryptographic hash function, and ∥ denotes concatenation. The natural differences in proteinoid electrical traits create randomness similar to traditional cryptographic random number generators. Our measurements show that Shannon entropy $H(K)$ is about 7.6 bits per byte for keys made from various proteinoid compositions. This is close to the theoretical maximum of 8 bits. The security of our system is a complicated math issue. It is a discrete multi-variable inverse problem. When we have the ciphertext $e_j$ and know the encryption algorithm, finding $k_i$ becomes difficult. We lack details about the proteinoid structure and its electrical properties. This makes the task as complex as recovering keys in standard symmetric ciphers. Our system resists replication attacks due to several complex layers in proteinoid synthesis and measurement. The proteinoid formation process involves complex polymerization dynamics that depend on numerous parameters:

$P\,(\text{composition, properties}) = f\,(T, t, [\text{amino acids}], pH, \text{catalyst}, RH)$

To synchronize the exact electrical properties, you need to control all parameters simultaneously, even if you know the amino acid composition. The electrical

measurement process follows specific protocols, including a frequency of 300 kHz, careful measurement conditions, equipment calibration, and precise contact placement and pressure. These factors serve as additional security parameters that must be replicated exactly. Proteinoid electrical properties change with different environmental conditions.

This behavior is described by the formula:

$C_i(T, RH) = C_{i,ref} \cdot (1 + \alpha_T \cdot \Delta T + \alpha_{RH} \cdot \Delta RH)$

where $\alpha_T$ and $\alpha_{RH}$ are sensitivity coefficients for temperature and humidity, respectively, specific to each proteinoid type. Furthermore, the electrical properties of proteinoid assemblies change over time according to:

$C_i(t) = C_{i,0} \cdot e^{-t/\tau_i}$ .

Here, $\tau_i$ is the time constant for each proteinoid composition. This temporal variation adds an extra layer of security: an attacker would need to precisely control synthesis, measurement, and environmental conditions to replicate our encryption keys, even if the proteinoid composition were known.

3.5 Key Reproducibility and Synchronization

We created a strong synchronization protocol. This helps reduce experimental variability and ensures reliable reproduction of key results. The sender and receiver use a calibration framework to handle measurement differences. The formula is

$C_{i,adjusted} = C_{i,measured} \cdot \frac{C_{ref}}{C_{i,ref}}$.

Here, $C_{ref}$ is the reference capacitance value set during key exchange. We implement a forward error correction algorithm that accounts for minor variations in electrical measurements:

$k_{i,corrected} = ECC(k_{i,measured})$.

This ensures that small variations in measurements do not affect the derived keys.

Electrical measurements are grouped into bins for better reproducibility. The formula is:

**$k_i = (\lfloor |C_i| \cdot 100/\delta \rfloor \cdot \delta \bmod 256)$ .**

**Here, δ is the quantization parameter found through experiments for the best results.**

**Every encrypted message has a sync header. This header has reference measurements that help the receiver calibrate their system:**

**header = E(sync pattern, $k_{ref}$)**

**The receiver can check if the key is correct by decrypting this header with their own key.**

**Both parties use environmental compensation factors from temperature and humidity sensors. The formula is:**

$$C_{i,compensated} = \frac{C_{i,measured}}{1 + \alpha_T \cdot \Delta T + \alpha_{RH} \cdot \Delta RH} .$$

**Our experiments show that this synchronization protocol reaches a key reproduction reliability of 98.7%. Our proteinoid-based cryptographic system works well in different measurement sessions. Our system maintains the key correlation needed for cryptography, and it also utilizes the special features of proteinoid assemblies. This boosts security against various types of attacks.**

**Reviewer #7: A process that cannot be reproduced in the same way and with the same initial values can be considered random, since it does not show determinism in its evolution. In contrast, a system that generates uncorrelated output values, but which can be replicated by knowing specific parameters of the generation algorithm, produces a pseudorandom sequence. From the description provided, the fundamental model proposed by the authors seems to be usable in systems with a random character. However, its applicability could be limited by the mechanism for distributing these data sets, called seeds in cryptography, essential in pseudorandom algorithms, where they are used as initial values to determine the behavior of the system. If the authors want to choose this way of generating cryptographic primitives, perhaps a study that would use the values generated for the seed of models derived from authenticated Diffie-Helmann would be more useful.**

**To clarify the sustainability of the proposed solution, especially in the context where, if functional, the proposed method would be an idea that should revolutionize the way cryptographic systems can be built, it would be useful to consider the following:**

1. A dedicated section describing the limitations of the method and describing possible mitigation methods.
2. Table 1 provides a comparison between different cryptographic technologies; however, for a more rigorous evaluation, it would be necessary to include quantitative criteria, such as system performance, security level, computational complexity or energy efficiency.
3. Clarify and rigorously detail the mechanisms by which proteinoids constitute a viable cryptographic solution, highlighting the aspects that give them security, robustness and practical applicability compared to traditional cryptographic methods.
4. Provide a detailed and rigorous analysis of the entropy and security level of the generated keys, highlighting the degree of randomization, resistance to cryptanalytic attacks and their compliance with current security standards.
5. A review of the abstract and introduction would be useful to explain more clearly, beyond a narrative presentation, how the proposed solution is built and how it works.
6. The completeness and synchronization of the key models and algorithms used by the sender and receiver.

Thank you for your review and suggestions for improving our manuscript.

We also agree that clearer explanations of its cryptographic foundations are important.

We want to point out that many of your concerns are similar to those from Reviewer #6. We have already addressed these by adding a new section called "Cryptographic Security Analysis" to the manuscript. This section includes detailed discussions on:

1. The way senders and receivers connect relates to your worry about "completeness and synchronization of the key models and algorithms" (point #6).
2. Mathematical security foundations offer analysis of our system's security. They improve on traditional methods. This approach addresses your points #3 and #4 about entropy, security level, and cryptanalytic resistance.
3. Protection against replication attacks shows the robustness discussed in point #3.
4. Key reproducibility and sync protocols tackle point #6 and the practical issues in point #3

For point #1, we added a subsection called "Limitations and Mitigations" in the Discussion section. This part talks about the current limits of our approach and suggests ways to address them.

### 4.1 Limitations and Mitigations

**Our proteinoid-based encryption approach shows promise, but we must recognize and address some limitations for practical use. The primary limitation of our current system is the relatively small key space (28) when using a single proteinoid sample. This offers weak security by today’s cryptographic modern standards usually require at least 128-bit security. We can mitigate this limitation by employing composite key generation. Here, we combine multiple proteinoid samples ($n \geq 16$) to create key spaces comparable to AES-128 or higher. Our early experiments with key composition show that this method works well, and it does not add much time to encryption or decryption. Environmental sensitivity is a significant challenge. The electrical properties of proteinoids change with temperature and humidity. Our experiments revealed capacitance variations of up to 15% when the temperature changed by ±5◦C, potentially disrupting key reproducibility. For field deployments, using controlled microenvironments protects proteinoid samples and shields them from environmental fluctuations.**

**Temporal stability is also a concern. Some proteinoid compositions change over time; for example, capacitance can shift by as much as 8% in just 30 days. Our strategy includes regular recalibration and the development of more stable proteinoid compositions. We add stabilizing agents, such as PLLA, to achieve this. Recent experiments with stabilized compositions have shown a drift reduction to less than 1% over the same period.**

**Manufacturing reproducibility poses challenges for scaling the technology. Current synthesis methods achieve about 80% reproducibility in electrical properties across batches, which is insufficient for dependable key generation. We are working on improved synthesis protocols and selection methods to find samples with similar**

electrical signatures. Advanced automated measurement systems have recently improved inter-batch reproducibility to 93%.

Key distribution remains a challenge. Our system currently requires a physical exchange of proteinoid samples. We are exploring ways to digitally encode proteinoid electrical signatures using secure multi-party computation protocols. This approach will help us establish keys remotely and safeguard our data against quantum attacks.

Computational efficiency is limited compared to optimized implementations of traditional cryptographic algorithms. Our current system takes about 3–7 ms for each operation, whereas AES operates in under 1 ms. Improving measurement methods and algorithms could greatly narrow this gap, although biological systems inherently operate on different time scales compared to electronic ones.

These challenges are significant, but they are not insurmountable for proteinoid-based cryptography. We are addressing each limitation with technological and algorithmic solutions. Early results are promising and point toward a path for practical use.

For point #2, we improved Table 1. It now has metrics for comparison.

These factors include:

- Computational complexity (operation counts)
- Security level (in bits)
- Energy efficiency (operations per joule)

Text added:

‘’

The computational complexity notation(e.g., \(O(n^2)\) describes how an algorithm's resource requirements scale with the input size \(n\). When the input size doubles, the computational needs grow approximately four times. This scaling behavior often shows

up in algorithms with nested loops. In these cases, the whole dataset is processed several times.

The security level, measured in bits (e.g., $70\text{--}128$ bits), quantifies cryptographic strength. To break a system with 128-bit security, an attacker would need to perform roughly $2^{128}$ operations. This number is so enormous that current technology makes achieving it almost impossible. A lower bound of 70 bits means weaker protection. This can make the system vulnerable to advanced attacks. Security can vary based on specific implementation details or methods.

Energy efficiency (e.g., $\sim 10$) ops/J (operations per joule). This metric shows how much computational work we perform for each unit of energy used. In resource-limited settings like mobile apps, power use is key. It has a significant effect on the app's performance. Higher values show better energy efficiency. Modern cryptographic systems strive to maximize this ratio while maintaining robust security levels.

Cellular automata-based encryption and our proteinoid-based method both scale linearly ($O(n)$). This makes them the most efficient choices in terms of computation. This linear relationship means that processing time increases with input size. So, performance stays predictable as data volumes grow. Chaotic systems have a complexity of $O(n \log n)$. In contrast, DNA-based cryptography runs at $O(n^2)$, and evolutionary algorithms are even more demanding at $O(n^2 \log n)$. These higher complexities can limit their use in large datasets or resource-limited settings.

Security levels vary considerably across these bio–inspired methods. DNA-based cryptography (70--128 bits) and cellular automata (80--120 bits) offer security similar to well-known cryptographic protocols. Chaotic systems provide moderate security, ranging from 60 to 100 bits. In contrast, evolutionary algorithms offer the lowest guaranteed security, with a range of 40 to 90 bits. Our proteinoid–based approach shows the widest security range (8--128 bits), reflecting its composition–dependent nature. The lower bound shows single-sample setups. The upper bound shows composite setups. These use multiple proteinoid samples to reach security levels that meet modern standards.

Energy efficiency demonstrates perhaps the most striking differences. Cellular automata–based encryption is the most efficient, with around $1000$ operations per joule. Next, we have chaotic systems that use about $500$ ops/J. Our proteinoid-based method follows at around $200$ ops/J. DNA-based cryptography uses roughly $10$ ops/J, and evolutionary algorithms need about $5$ ops/J. This difference is due to their complex biochemical and iterative computing needs. This parameter matters a lot for IoT devices, embedded systems, and other energy-limited settings.

Our proteinoid-based cryptography shows a strong balance. It has linear computational complexity, adjustable security levels, and moderate energy efficiency. Cellular automata are more energy-efficient now. However, our method may offer better adaptability and quantum resistance because it's based on biology. Plus, there's still room to improve as the technology develops."

- We appreciate your suggestion regarding the potential application of our system to seed generation for authenticated Diffie-Hellman protocols. This is an excellent direction for future work that we have now mentioned in our expanded Discussion section.

Text added:
''Proteinoid assemblies have unique electrical properties. This makes them great for creating high-entropy seeds. These seeds are useful for key exchange protocols. A key use is in authenticated Diffie-Hellman protocols~\cite{barker2017recommendation,alvarez2017algorithms}. Here, the system's security relies on how unpredictable the random number generation is. Proteinoid assemblies show natural entropy and unique electrical signatures. These traits may offer a biological source of randomness that resists algorithmic prediction. Using proteinoid-derived values for key generation in Diffie-Hellman exchanges can help resist quantum computing attacks. This helps protect against threats to purely mathematical methods. Our early research shows that proteinoid-based seed generation has Shannon entropy values near the theoretical maximum. The autocorrelation profiles do not show any clear patterns across different sampling intervals. This hybrid method combines the natural randomness of proteinoid systems with reliable cryptographic protocols~\cite{borisov2019adaptation,barker2018recommendation}. It shows promise for future research. This approach could improve security while keeping key exchange systems compatible."

**Table 1.** Comparison of Biological Encryption Technologies. This table summarizes various bio-inspired encryption methods, highlighting their features, advantages, and limitations. DNA-based cryptography uses DNA's high information density and parallel processing for secure communication. Cellular automata encryption employs the dynamic properties of these systems, inspired by biological cells, to create complex patterns. Chaotic systems, mimicking unpredictable biological processes, are used in cryptography due to their sensitivity to initial conditions. Evolutionary algorithms, inspired by biological evolution, aim to enhance cryptographic methods. However, these methods face challenges like scalability, implementation issues, and the need for more research to ensure security. The proposed proteinoid-based cryptography in this paper aims to utilize the unique properties of proteinoid assemblies—self-organization and molecular recognition—to create a new, secure encryption system.

| Technology | Key Features | Advantages and Limitations |
|---|---|---|
| DNA-based cryptography [13] | - Exploits high information density of DNA - Parallel processing capabilities - Molecular-level encryption - Computational complexity: $O(n^2)$ - Security level: 70-128 bits - Energy efficiency: ~10 ops/J | - High storage capacity - Potential for DNA computing - Challenges in practical implementation - Susceptible to errors and mutations |
| Cellular automata-based encryption [23] | - Discrete models inspired by biological cells - Dynamic behaviour and complex patterns - Local interactions and global emergence - Computational complexity: $O(n)$ - Security level: 80-120 bits - Energy efficiency: ~1000 ops/J | - Parallel and distributed processing - Generates complex encryption patterns - Scalability and computational efficiency - Limited research on cryptanalytic resistance |
| Chaotic systems [23] | - Exhibits unpredictable and random-like behaviour - Sensitive to initial conditions - Inspired by chaotic processes in biology - Computational complexity: $O(n \log n)$ - Security level: 60-100 bits - Energy efficiency: ~500 ops/J | - High sensitivity to initial conditions - Generates random-like sequences - Potential for secure communication - Requires careful parameter selection |
| Evolutionary algorithms [24] | - Inspired by biological evolution - Optimization and adaptation mechanisms - Population-based search and selection - Computational complexity: $O(n^2 \log n)$ - Security level: Variable (40-90 bits) - Energy efficiency: ~5 ops/J | - Optimizes cryptographic primitives - Adapts to changing security requirements - Computationally intensive - Convergence to global optimum not guaranteed |
| Proteinoid-based cryptography (proposed) | - Self-organization and molecular recognition - Vast key space and dynamic behaviour - Adaptive and context-aware encryption - Computational complexity: $O(n)$ - Security level: 8-128 bits (composition dependent) - Energy efficiency: ~200 ops/J | - Unique properties of proteinoid assemblies - Potential for lightweight and energy-efficient algorithms - Self-organizing and self-authenticating systems - Experimental validation and further research required |

**New Abstract**

We present an innovative cryptographic technique inspired by the self-assembly processes of proteinoids—thermally stable proteins that form spontaneously under prebiotic conditions. By emulating the deterministic yet complex interactions within proteinoid assemblies, the proposed method generates secure encryption keys and algorithms. We measure the unique electrical properties of proteinoid microspheres. Their capacitance values range from $-656.6$ to 434.9 nF. Then, we convert these measurements into encryption keys using the formula $k_i = (\lfloor |C_i| \cdot 100 \rfloor \mod 256)$. The approach harnesses the inherent unpredictability of proteinoid behavior to create a robust and adaptable encryption framework resilient to cryptanalytic attacks. The encryption process uses modular multiplication: $e_j = (m_j \cdot k_i) \mod 256$. This changes plaintext into ciphertext. The security relies on electrical signatures that depend on the composition. Experimental results show that this bio-inspired system aligns with contemporary encryption standards, offering significant benefits in key generation and distribution. Our implementation has a linear computational complexity of $O(n)$. It offers security levels ranging from 8 to 128 bits, based on composition. Additionally, it is energy efficient, performing about 200 operations per joule. Statistical analysis further affirms the high randomness of the generated keys, highlighting the potential of biological processes in advancing cryptographic security.

**Text added in Introduction**

**‘’Our cryptographic framework works in a structured way. It uses the electrical properties of proteinoid assemblies. We start by making proteinoid microspheres. This happens through thermal polymerization of amino acids at 180±1°C. Next, we check their electrical properties. We measure resistance (R), impedance (Z), and capacitance (C) with precision LCR meters at 300 kHz. The capacitance values range from -656.6 to 434.9 nF. These values depend on the amino acid composition. They are used to generate cryptographic keys with the formula ki = (⌊|Ci| · 100⌋ mod 256). Encryption occurs via the function ej = (mj · ki) mod 256, where mj represents plaintext ASCII values. This procedure creates an encryption system that is both predictable and unpredictable. Its security comes from the complex interactions of molecules in the proteinoid structures.We use several evaluation metrics: computational complexity**

**(O(n)), security level (8-128 bits based on composition), and energy efficiency (about 200 operations per joule). This makes our method a biologically-inspired option compared to regular symmetric encryption algorithms."**

---

7. PLOS authors have the option to publish the peer review history of their article (what does this mean?). If published, this will include your full peer review and any attached files.

If you choose "no", your identity will remain anonymous but your review may still be made public.

**Do you want your identity to be public for this peer review?** For information about this choice, including consent withdrawal, please see our Privacy Policy.

Reviewer #6: No

Reviewer #7: No

---

[NOTE: If reviewer comments were submitted as an attachment file, they will be attached to this email and accessible via the submission site. Please log into your account, locate the manuscript record, and check for the action link "View Attachments". If this link does not appear, there are no attachment files.]

While revising your submission, please upload your figure files to the Preflight Analysis and Conversion Engine (PACE) digital diagnostic tool, https://pacev2.apexcovantage.com/. PACE helps ensure that figures meet PLOS requirements. To use PACE, you must first register as a user. Registration is free. Then, login and navigate to the UPLOAD tab, where you will find detailed instructions on how to use the tool. If you encounter any issues or have any questions when using PACE, please email PLOS at figures@plos.org. Please note that Supporting Information files do not need this step.
