# Supplementary material for: Bio-inspired cryptography based on proteinoid assemblies
Source: PLoS One. 2025 May 28;20(5):e0324761. doi: 10.1371/journal.pone.0324761 (PMC12118996; doi:10.1371/journal.pone.0324761)
Supplement: Attachment — Submitted filename: Recommendation.docx [file pone.0324761.s002.docx]

Dear Hunyadi,

Thank you for your invitation.

Although this manuscript (PONE-D-24-49897R1) constructs a bio-inspired system utilizing simulated proteinoid interactions, the main content still belongs to the biological discipline. This caused me some trouble in reviewing the manuscript.

Although from the biological point of view, this article embodies a certain research value. However, from the perspective of cryptography, I think the author has some improvements to make, and make some comments on the content of the article.

Here are my opinions:

1. In Section Ⅰ "Introduction", the author stated "Bio-inspired cryptography has emerged as a significant area of research, using the complex and distinctive characteristics of biological systems to create advanced encryption and decryption methods [1–10]." Although the author wants to express the development of this cryptographic primitive, it is recommended that the author make a classification or comb of these references rather than simply cite them.

2. The author cites Figures 1-13 throughout the manuscript, but does not present specific figures. The author is responsible for the integrity and readability of the manuscript.

3.In Figure11, the author claims “The different times to encrypt and decrypt may boost security.” For symmetric encryption, the difference in encryption and decryption time is also common. This is especially true for asymmetric encryption. This conclusion does not seem to be accepted in the cryptography. Please give a specific explanation of cryptography theory.

And, in the Abstract, the author claims that "It is also extremely resistant to attacks from both classical and quantum computers." At present, cryptographic methods that can resist quantum attacks are also the focus of research. We do not deny the achievement of the authors, but please should provide standard security proof.

4.In Figure12, the author claims “Using proteinoid structures in the algorithms adds security and complexity.” The author also states that it increases the computational complexity, then why use this encryption method?

5. In this paper, the author uses symmetric encryption cryptosystem to build a bio-inspired system. However, it seems that the author does not explain the encryption and decryption method and the generating process of the key in detail, which is not conducive to the understanding of the article.

6. When referring to Figures and Tables in the paper, we strongly recommend that authors give specific and explicit statements, but this does not mean meaningless repetition. The author should carefully revise the language expression of the full text.

It is an honor to help your journal complete the task of review, and we appreciate your journal's rigorous scientific attitude and fast review speed. At the same time, we also ask you to pay attention to our submission (PONE-D-24-59581) entitled 'Dynamic Multi-key FHE under new working mode from LWE'.

Best wishes

Prof. Yang

8 January 2025
